# Differences between acute coronary syndrome with st elevation according to be ischemic or non-ischemic cause in a cardiological ICU

**DOI:** 10.1186/2197-425X-3-S1-A544

**Published:** 2015-10-01

**Authors:** FM Acosta, O Moreno, AM Pérez, M Muñoz, M Colmenero, L Peñas

**Affiliations:** Hospital San Cecilio, Granada, Spain

## Introduction

Is established the benefits of early reperfusion in ST elevation myocardial infarctions and there is a program of primary percutaneous coronary intervention (PCI) to goal the soon opening of the coronary artery affected. Its common the presence of ST elevation caused by conditions other than acute ischemia (no ischemic ST elevation, NISTE) which may be confused as STEMI.

## Objectives

To know epidemiology aspects and differences between patients with Acute Coronary Syndrome (ACS) with ST elevation according to cause ischemic or not, in patients attended in a Cardiological ICU during a year.

## Methods

Consecutive registry of patients seen in a year (15/02/14 to 15/02/15) in a cardiological ICU, whom were approached as ACS with ST elevation, based on Spanish ARIAM (Acute Myocardial Infract Delay Analysis) database.

## Results

There were included 146 patients with the suspect of ASC with ST elevation, 111 men (76%) and 35 women (24%) with a medium age of 62.5 y.o.; they were discharged with final diagnostic as STEMI 121 patients (82.9%) and NISTE 25 patients (17.1%) between figure coronary spam 7 cases (28%), myopericarditis 2 (8%), transitory ST elevation 5 (20%), LBBB, LVH, High blood pressure event associated, early repolarization each one with 2 cases (8%) and other cases 3 (12%). Respect of type of catheterism these were primary PCI 110 cases (75.3%), rescue catheterism 2 (1.4%), early catheterism 20 (13.7%), finding a total of 17 cases with normal coronary or without significant injury; in the group of NISTE were performed 18 catheterism (10 primary and 8 early) finding normal cases in 16. Respect the age it was similar in both groups with medium age of 62.98 (STEMI) and 60.5 y.o. (NISTE). The proportion of men/women was higher in STEMI group (78/21 against 64/36 in NISTE group). The stay in ICU was minor in NISTE group (1.72 against 2.35) but something higher in cardiological hospitalization service(4.08 against 3.57), with similar hospitalstays in both groups. The mortality was 5.5% (8 cases, all in the STEMI group) with a medium age of 81 y.o.

## Conclusions

Is common the presence of other diagnostic as NISTE in approach and treatment of STEMI. The age was similar in both groups, being men proportion higher in STEMI group. The hospitalary stay was similar in both groups, but higher in cardiology hospitalization service of NISTE group.

